# A Design Thinking Approach for Transnational Adaptation of 2 Mobile Mental Health Apps: Tutorial for Researchers and Practitioners

**DOI:** 10.2196/77048

**Published:** 2025-09-17

**Authors:** Sylvie Bernaerts, Tom Van Daele, Christian Korthé Carlsen, Søren Lange Nielsen, Jolanda Schaap, Yvette Roke

**Affiliations:** 1 Psychology and Technology, Centre of Expertise Care and Well-being, Thomas More University of Applied Sciences, Campus Sanderus, Molenstraat 8, Antwerp, 2018, Belgium, 003234324050; 2 Centre for Digital Psychiatry, Region of Southern Denmark, Odense, Denmark; 3 Expertise Center for Autism Spectrum Disorder, GGz centraal, Almere, The Netherlands

**Keywords:** digital mental health, design thinking, user participation, end-user involvement, transnational upscaling, mobile phone apps

## Abstract

Digital mental health solutions have great potential to enhance mental health care. However, barriers at the level of users, interventions, and context hinder engagement and uptake. Involving users in the design, adaptation, and implementation process has been put forward as a potential solution; however, instructions and examples on how to do so are limited. One potential framework is design thinking. Although design thinking is a common approach in the business community, its use for guiding development and adaptation processes is not yet a common practice in the context of digital mental health. Unsurprisingly, it is difficult to find concrete instructions on how to do this, even more so in an international context. Therefore, the SUPER (Successful User Participation Examples and Recommendations) project aimed to develop guidelines for entrepreneurs and mental health organizations on how to involve end users and mental health care professionals in the transnational development, implementation, and adaptation of mental health technology. This paper describes the design thinking process that led to those guidelines and how these can be undertaken by researchers, practitioners, or developers in the context of digital mental health. The process is illustrated with 2 adaptations of digital mental health solutions following this approach, executed by the SUPER consortium in the Netherlands and in Denmark. The learnings from these 2 pilots are provided in the form of key considerations and highlights of issues experienced during both design thinking processes. The overall aim is to guide practitioners, developers, and researchers toward better development and international adaptation of digital mental health.

## Introduction

### Background

Over the past 2 decades, there has been increasing research evidence that digital mental health interventions can be effective in supporting people’s mental health in a variety of conditions [1] and across age groups, such as in the older individuals [2], adults [3], and youth [4]. Perhaps unsurprisingly, the global mental health app market has been rapidly growing, from an estimated market size of US $6.25 billion in 2023 toward an expected US $17.52 billion in 2030 [5]. One of the main drivers is the hope that digital mental health can be a means to increase accessibility, affordability, and overall effectiveness of mental health care. There is, however, more to say about the potential of mental health apps than projected market share. As dissemination of digital mental health solutions started, their proven efficacy did not seem to easily translate to effectiveness in everyday life. One of the main challenges, especially when apps are being implemented outside of routine care settings and without clinician support, relates to low adherence and high dropout. Retention of self-guided mental health app usage by the general population, for example, turns out to be low, with a median 15-day retention of 3.9% and 30-day retention of 3.3% [6]. Also in clinical populations, similar trends have been reported for mental health app usage without clinician support [7], with high dropout rates from the very onset of using an app.

Research has consistently shown 3 levels of factors influencing user engagement with digital mental health. These factors include user-related, program-related, and technology- and environment-related factors [8]. User-related constructs include users’ demographics, such as age, gender, personality traits, and mental health symptoms. Program-related factors involve the intervention’s content, perceived fit and usefulness, level of guidance, social connectedness, and impact. Finally, the technology itself or its implementation, such as technical issues, privacy and confidentiality, social influence, and training, can also have an impact.

Similarly, but specifically for children and young people, user engagement with digital mental health interventions has been found to be impacted by intervention-specific factors, such as suitability, usability, and acceptability, and person-specific factors, such as motivation, opportunity, and capability [8]. More recently, Zhu et al [9] also described barriers and facilitators to digital mental health interventions, in particular, for depression, anxiety, and stress in adolescents and young adults on the same 3 levels, namely, individual factors, such as needs, perceived benefits and risks, and motivational challenges; interventional factors, such as quality, design, and content; and external-level factors, such as integration within existing programs, social norms, and marketing.

Since user engagement with digital mental health interventions is significantly influenced by both person- and intervention-specific factors, one possible solution to make interventions more engaging for users is to involve them and other stakeholders in the actual design process [10-12].

Digital mental health solutions, such as mobile apps, virtual and augmented reality, web-based platforms, or wearables, have often been created either by companies, by researchers, or by mental health care professionals, usually based on some evidence-based principles, often cognitive behavioral therapy. On the one hand, these solutions have most often been developed for intended users rather than with them, eventually leading to unappealing products that are ill-adapted to or not well integrated into existing health care systems. On the other hand, there are also beautifully designed solutions that unfortunately lack an evidence base.

Fortunately, over the past decade, there has been a growing research trend toward including end users and other stakeholders in the design and development process of digital mental health solutions in order to tackle the aforementioned barriers and to create better, more efficient, and more engaging digital mental health solutions. In these studies, a variety of concepts referring to human- or user-centered design have been used, for example, user-centered design, co-design, or design thinking.

In short, these concepts are all focused on involving users in iterative product or service design in order to assess their needs and the usability of the product or service; however, the extent to which users have control over design decisions varies across the different concepts. For example, the user-centered design approach emphasizes user involvement to improve the understanding of both user and task requirements, as well as the iteration of design and evaluation. In particular, users are involved in usability testing, but they primarily serve as consultants [13-15]. Co-design prioritizes collaboration with users so that users actively participate in the design process together with the trained designers. In this respect, users serve as cocreators and are considered partners throughout the process from need exploration to idea generation and beyond [14]

Finally, design thinking is, rather than a design method, a more creative innovation framework and way of thinking and addressing challenges that is focused on empathy and users’ needs and uses various methods from design approaches and differing levels of user involvement to meet those needs [15]. The most important idea, however, remained consistent, namely that different stakeholders were to be consulted and involved throughout the design process from idea conception to implementation.

A clear summary and description of the history behind and similarities and differences between different human-centered design approaches have already been reported by Vial et al [15]. With their review, it was also found that integration of human-centered design methods in the development of digital mental health solutions is, nonetheless, still rare and relies too little on designers or design research. Although multiple studies in the field of digital mental health do report adoption of participatory design, co-design, user-centered, or other specific design approaches, most researchers seem to have failed to adequately define and explain their approach and how it impacted their design process [15].

In addition, involving stakeholders in the design process does not necessarily create more effective solutions. For example, reviews on user involvement in the design of youth digital mental health interventions [16,17] found that due to lacking outcome data, there was limited evidence for user involvement leading to routine uptake of technology-based youth mental health and well-being solutions in practice or more effective interventions; however, it did aid in designing more relevant, accessible, and usable interventions. In contrast, a more recent review revealed that user involvement in the design process can indeed enrich ideas and enhance digital mental health interventions’ cultural sensitivity, acceptance, and engagement [18].

One of the aforementioned approaches to involve users in the digital mental health design and implementation process is design thinking [15]. Although design thinking is a common approach in the business community, its use for guiding development and adaptation processes in mental health care is not yet a common practice. Although the idea and potential of applying design thinking for digital mental health design and implementation is not new [15,19], it is nevertheless difficult to find concrete guidelines on how to do this, even more so on how to do so in an international context. Therefore, the SUPER (Successful User Participation Examples and Recommendations) project [20] aimed to develop guidelines for entrepreneurs and mental health care organizations on how to involve end users and mental health care professionals in the transnational development, implementation, and adaptation of mental health technology.

### The SUPER Project

This project was funded by the Interreg North Sea and relied on a collaboration of 3 organizations: GGz Centraal, the Centre for Digital Psychiatry, and Thomas More University of Applied Sciences. GGz Centraal is the fourth-largest psychiatric hospital in the Netherlands and offers specialized treatment to people with mental health problems in all phases of their lives by providing diagnostics, treatment, counseling, prevention, and information to people with psychological, psychiatric, or psychosocial problems. GGz Centraal strives to offer care as close to their clients as possible. In this respect, coauthor YR (psychiatrist) and her team have developed the digital mental health app SAM (Stress Autism Mate) [21], together with their clients. SAM is a personalized app that supports the self-management of clients with an autism spectrum disorder in dealing with their daily experienced stress.

The Danish Centre for Digital Psychiatry is a frontrunner in the use of digital solutions to promote mental health and psychiatric treatment and is part of the Mental Health Services in the Region of Southern Denmark. The goal of the Centre is to ensure that all Danes have easy and equal access to mental health and psychiatric treatment through digital psychiatry. In this project, they collaborated with Prof. Lene Berring, who developed the Danish SAFE app [22]. It is an app intended to support people who self-harm, their family members, and their health care providers [23,24].

The research line Psychology and Technology, at the Thomas More University of Applied Sciences, conducts applied, practice-oriented research in the field of psychology and technology. There is a strong focus on the interaction between both domains and the synergy they create, in particular within mental health care and human-technology interaction.

With the SUPER project, this consortium aimed to create guidelines on how to involve users in the transnational adaptation of mental health solutions. To do so, we set up evidence- and experience-based guidelines and tested them in real life with existing mobile apps. In the following sections, we describe the design thinking process for the international adaptation of 2 mobile mental health apps. The Dutch SAM app was adapted to fit the Danish context, and the Danish SAFE app was adapted to suit the Dutch context.

### SAM App

**Figure 1.**
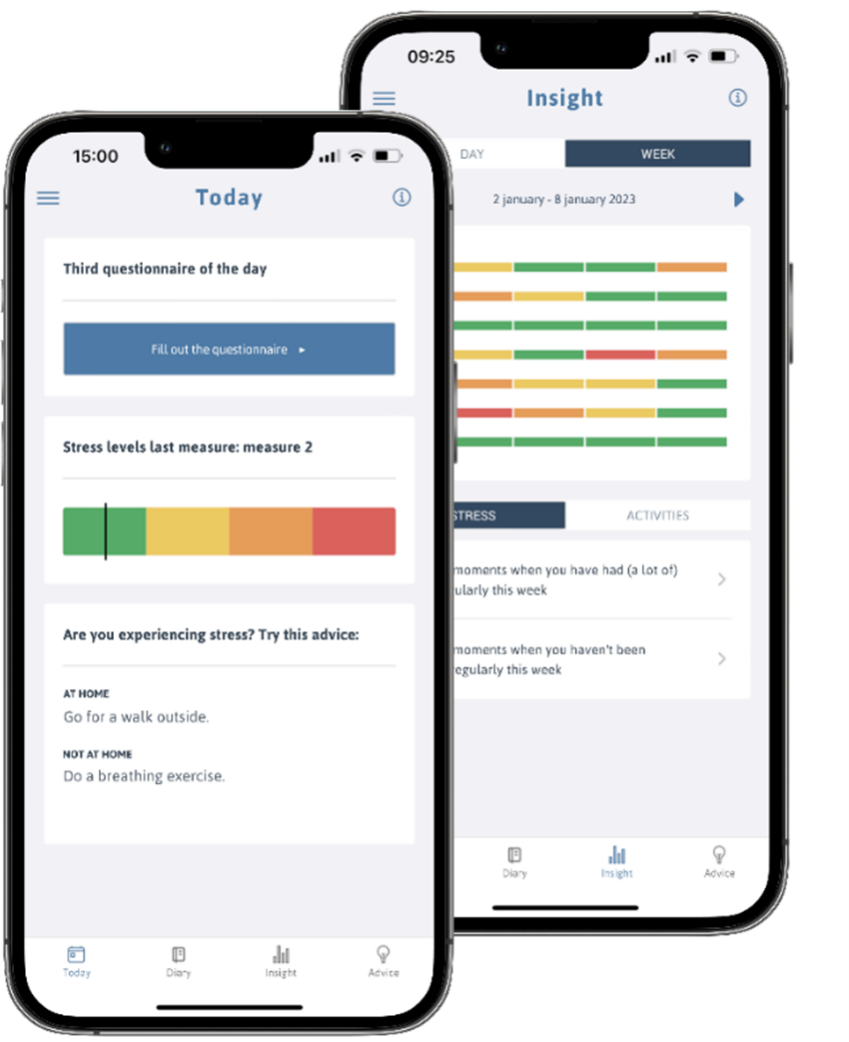
Screenshots of the Stress Autism Mate app.

Stress Autism Mate (SAM) is a mobile app cocreated by mental health researchers, mental health practitioners, and individuals with autism [25] (Figure 1). SAM aims to support individuals with autism in recognition and self-management of stress in daily life. To do so, the app assesses the user’s perceived stress level 2-4 times per day by means of a short questionnaire. Consequently, the app provides general and personalized coping advice depending on the user’s current stress level. Moreover, an overview of the perceived stress levels can be found on the “Insight page” of the app, available at the daily and weekly levels. As such, the app also provides insights into the user’s own stress levels over time. Initial studies with the SAM app have shown that the app can help to reduce stress and improve quality of life in individuals with autism [25,26]. The SAM app collects personal data (email, mental health questionnaire scores) via a password-protected user account. A section on the terms of use is available in the app, also referring to the privacy statement detailing data collection and data processing.

### SAFE App

**Figure 2.**
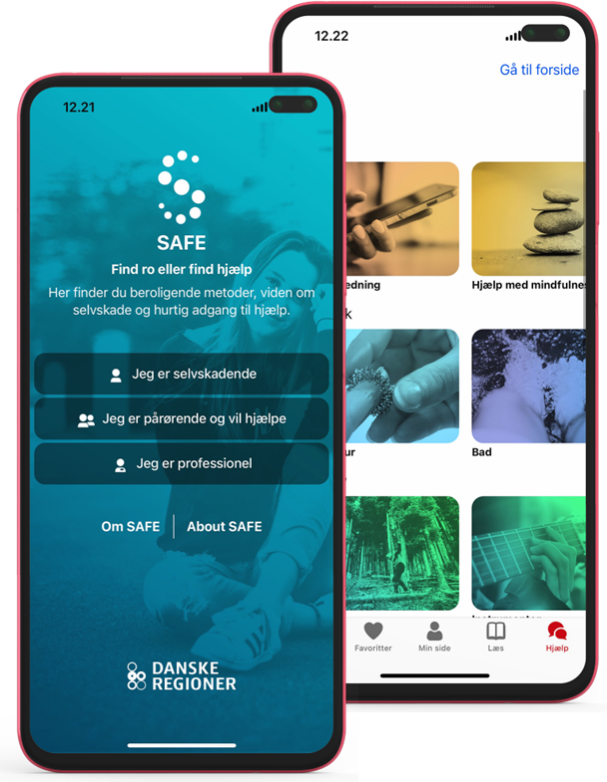
Screenshots of the SAFE app.

SAFE is a Danish mobile app intended to both help and inform people who self-harm, their relatives, and health care practitioners [24] (Figure 2). SAFE aims to empower its users in decision-making, seeking distraction to prevent or divert self-harm, and supports interactions, dialogues, and experience sharing between the different target groups. The SAFE app was cocreated with people with lived experience of self-harm and is based on the Safewards theoretical framework [27] and Trauma-Informed Care [28]. Although the SAFE app’s effectiveness is still being studied, preliminary results have shown that SAFE can mitigate negative experiences for patients admitted to hospital after self-harming, as they found the provided calming methods helpful and distracting. In addition, SAFE was perceived as a positive and caregiving supplement in treatment as usual [29]. At the request of the users in the cocreation process, the SAFE app is used individually and does not store personal data.

### Aims

With this tutorial, we aim to provide researchers, practitioners, and developers a step-by-step guide to adapt an existing digital mental health app for use in another country, based on the design thinking framework and illustrated by 2 examples implemented by the SUPER consortium. We provide concrete design thinking steps and detailed actions in a context of involving users in the international upscaling of 2 mental health apps, the Stress Autism Mate (SAM) and the SAFE app. Examples of activities and methods that we have adopted in our own design thinking process are provided, and lessons learned from this process are described. This tutorial thus provides an overview of the design thinking methodology, illustrated with 2 concrete examples to guide user involvement.

## Design Thinking Methodology

### Overview

Design thinking is defined as a method of meeting people’s needs and desires in a technologically feasible and strategically viable way [30] and was originally mostly known in the business community. Design thinking has found its way to various sectors, including health care, though implementation is not yet common. In practice, design thinking is considered a mindset for problem solving, and it provides a framework for designing practical products that truly answer different users’ specific needs and desires [19]. Design thinking helps to involve different stakeholders, in particular the intended users, from the start of the design process, ensuring the product meets their needs and challenges. This can be especially relevant when adapting solutions to new languages and cultures.

Early involvement of users also helps to quickly test assumptions and challenges to avoid lengthy and, at times, indifferent development processes. However, design thinking is neither a linear nor is it a one-size-fits-all approach. Design thinking involves 5 steps to guide the user participation process but does not follow one strict protocol [30].

The following 5 steps can overlap and should be considered as an iterative process. The first step is to empathize, that is, getting to know the users by understanding their experiences, challenges, and needs. The second step is to define the challenges and needs that a digital solution should address, based on the insights gathered in the prior phase. The third phase, ideate, focuses on brainstorming and generating creative solutions to your defined challenges and needs. This is followed by a prototype, meaning that one or more simple versions of the solution are created and shared with potential users for feedback. In the final phase, the test phase, users interact with the refined prototype to gather their feedback to further refine the solution. It is possible to go back and forth between the phases to create digital mental health solutions that are effective, meaningful, culturally relevant, and user-centered.

In an ideal scenario, digital mental health solutions for certain target groups are co-designed with these target groups and other relevant stakeholders. However, in the context of transnational adaptation of an already existing mental health mobile app, starting from scratch is often impossible due to timing or monetary constraints. It is, nevertheless, still important to involve users in the adaptation process to ensure that the solution actually adheres to the intended users’ needs and context. In the following sections, we therefore describe examples of how we operationalized the design thinking steps in a manner that was feasible for mental health care organizations. All study documents and qualitative data can be retrieved on the Open Science Framework [31].

### Ethical Considerations

After review by the internal Institutional Review Board at the Centre for Digital Psychiatry and at GGZ Centraal, informed consent procedures were stated to be sufficient, and formal ethical approval was not deemed necessary, as the study was not considered to be a medical study. Prior to data collection, participants were informed verbally and by means of an information letter about the study objectives, study design, and data processing, and all participants provided written informed consent. Participants were not compensated for their participation in the study. When working with vulnerable groups, such as individuals with autism, it is also important to be mindful of ethical concerns and create a respectful and safe environment for their participation. To accommodate the preferences of the participants, interviews were held in different locations, including their homes, a local activity center, residential care facilities, and at the Centre for Digital Psychiatry. This flexibility ensured that participants felt comfortable and could provide genuine feedback in environments familiar to them. Moreover, in the context of digital mental health, privacy and data protection actions also warrant additional attention. Data were processed anonymously, meaning no personal identifiable information was kept by the authors. All project data made available via the Open Science Framework is anonymous. No images or identifiable materials were used in the paper or MultimediaAppendices 1  2.

### Step 1: Empathize

The empathize phase focuses on understanding intended users’ needs and challenges by observing, listening, and asking questions. In the context of mental health, the various stakeholders include, for example, patients, mental health care practitioners, family members, developers of mental health apps, mental health care organization directors or managers, and even health policymakers. Key activities in this phase initially consist of desk research, surveys, interviews, and focus groups in order to learn about the already existing mental health solutions on the market but mainly to identify the challenges and needs of the intended users of digital mental health solutions. These activities help to determine the actual need for the solution and to challenge underlying assumptions about the matter. In the context of digital mental health, it is also essential during this phase to investigate the necessary monetary investments and the legal and regulatory requirements, such as data protection and medical device regulations, to ensure local compliance.

#### Desk Research

##### SAM App

In order to adapt the Dutch SAM app for use in Denmark, we used a mixed methods approach to gather insights from clinicians and individuals with autism (the intended users). This helped us to understand both clinical and user perspectives related to the SAM app. To gain insight into the needs and challenges of individuals with autism in Denmark, in particular concerning stress, we first conducted desk research. In practice, this entailed a Google search and different forums as well as books on the subject, seeing as the health care organizations without access to scientific sources would do the same. In addition, we also looked for existing digital solutions for stress in autism but found none.

##### SAFE App

Similarly, to adapt the Danish SAFE app for use in the Netherlands, we used a mixed methods approach, including desk research, interviews, and (remote) usability tests to gather insights from clinicians and individuals who self-harm and their relatives (eg, parents or siblings). This approach helped us to understand both clinical perspectives and those of individuals who self-harm and their family and friends. To gain insight into the needs and challenges of individuals who self-harm in the Netherlands, in particular concerning online guidance and help, we first conducted desk research. In practice, this entailed an internet search on both social media and a scientific literature database (ie, PubMed) on the subject of digital mental health solutions for individuals who self-harm. In addition, we also discussed the topic with clinicians with expertise in the matter at GGz Centraal and at Karakter, a Dutch academic institute for child and youth psychiatry.

### Stakeholder Engagement

#### SAM App

To gain deeper insight, we also interviewed both clinicians and individuals with autism about their needs concerning tackling stress and about existing solutions to tackle stress, both digital solutions (eg, apps), and other methods (eg, relaxation exercises). To do so, we first reached out to multiple patient organizations, including nongovernmental organizations and their local departments, public organizations working with individuals with autism (eg, regional treatment offers and municipalities), and mental health care organizations, explaining to them about the objective of the SUPER project as well as the specific aim of the interviews and focus group. These organizations were either contacts or partners (ie, the Region of Southern Denmark) or were found through the initial desk research. For the interviews, we initially recruited clinicians working with individuals with autism, as this provided easily accessible and valuable input on the intended users. In this way, we were able to learn more about this vulnerable target population before reaching out to them directly. Specifically, we started with a group interview involving 3 clinicians who work with the intended users to gather expert opinions on the need for and potential impact of the SAM app.

We also interviewed 6 individuals with autism, separately. These participants ranged in age from teens to people in their fifties. Each interview lasted between 50 and 90 minutes. These interviews also explored the need for the SAM app in Denmark and captured various experiences and challenges related to stress in daily life. For example, by asking questions such as “Do you experience any challenges with stress in your everyday life?,” “How do you deal with these challenges?,” “Which tools do you have access to?.” To accommodate the preferences of the participants, these interviews were held in different locations, including their homes, a local activity center, residential care facilities, and at the Centre for Digital Psychiatry. This flexibility ensured that participants felt comfortable and could provide genuine feedback in environments familiar to them.

#### SAFE App

Consistent with the SAM process, we also planned to interview clinicians, individuals who self-harm, and their family and friends about their needs concerning self-harm. To do so, we first contacted colleagues and reached out to department heads within our own professional network via email, namely at GGz Centraal and Karakter, explaining to them about the objective of the SUPER project as well as the specific aim of the interviews and usability tests. We gave 3 presentations about the SUPER project, its objectives, and the tandem pilots, of which 2 were in-house at GGz Centraal and 1 was during a video conference call meeting with interested professionals at Karakter. It was thus quite time-consuming to recruit mental health care professionals to be involved in the adaptation process of the SAFE app, even with a large network within mental health care. For the interviews, we first recruited clinicians in order to learn more about self-harm in general, the current therapeutic approaches, and the role of digital mental health therein and to get feedback on initial prototypes of SAFE NL. Specifically, we conducted 4 consecutive remote focus groups with 7 clinicians via conference call using Microsoft Teams, that is, 6 psychologists and 1 psychiatrist, who work with individuals who self-harm. We opted for remote sessions via video conference call, as was preferred and deemed most practical by the participants. These focus groups lasted approximately 60 minutes each and were moderated by 2 project members (YR and JS), who shared the responsibilities of note-taking and moderating. The first session aimed to gather expert opinions on the need for and potential impact of the SAFE app. The clinicians were also asked about their experiences with mobile mental health apps. To do so, we used an interview guide, including questions, such as “Do you have experience with using mobile mental health apps in the context of self-harm?” After we provided examples, we also asked “What is not yet named and important to take into account?,” “When is a pilot successful for you?” and “Which barriers and facilitators do you envisage?”

### Step 2: Define

The define phase focuses on analyzing the findings gathered during the empathize phase, clearly identifying and defining the scope of the application, along with intended users and other stakeholders and their needs, goals, and challenges, as well as clearly defining potential problems. This helps to ensure that the solution is aligned with the intended users’ needs and also allows us to identify the necessities toward integration in existing services. One interesting method is the use of personas, which are fictional, summarizing descriptions of an individual based on real data. They are general enough to represent a stakeholder group but specific enough to allow the design team to focus on specific users' goals and needs [32]. Personas can be a good starting point for summarizing the findings from the empathize phase and defining the intended users and their needs, goals, challenges, and potential cultural differences. Another common method is mapping out the user journey and potential workflow when using the solution. Visualizing the users’ experience as they interact with the product aids in highlighting pain points, opportunities, and possible improvement areas, in turn helping to better understand the user’s perspective. Once stakeholders and their needs have been determined, inviting them into the defining step via workshops or focus groups can be beneficial and empower users by giving them a voice in the design process.

#### Persona

##### SAM App

Based on desk research and the interview, we created a persona to articulate the goals, challenges, needs, and dreams of a person with autism in Denmark in relation to the SAM app (Table S1 in Multimedia Appendix 1). The persona helped us to get a better understanding of the target group, as it was, for the Danish project partners, a new group to work with. This persona was used to prepare the interview and usability test with individuals with autism.

##### SAFE App

No persona was made for the users of the SAFE app.

### Step 3: Ideate

The ideate phase is focused on exploring possible directions or strategies for the solution before narrowing down to those that seem most promising and meet the intended users’ defined needs and challenges. Ideation involves a team effort. Whether a classic brainstorm or a mind map with different stakeholders, these methods provide an excellent starting point for exploring ideas and meeting the defined needs and challenges. Another possible method is “How Might We.” This technique entails converting challenges into questions starting with the words “How might we” [32]. For example, the challenge “Some individuals cannot relate to the content in the app,” can be rephrased as “How might we create an inclusive application?” In the ideate phase, meeting with developers to discuss technical possibilities can also be beneficial, whether adapting an existing solution or developing a new one. Moreover, it helps to prepare an action plan detailing how your solution can be adapted to and tested with the intended user in the new market. Even when adapting existing solutions, the ideate phase plays a minor but crucial role. While the overall concept of the digital solution is already established, this process can help to look at the solution from new perspectives. It can also provide opportunities for incremental improvements and new features within the limits of the existing solution and potential financial restraints.

#### SAM App

As an example of the iterative process, based on the interviews, tests, and observations during the test with both clinicians and individuals with autism, we explored and identified a set of recommendations, which could improve the SAM app for use in Denmark (Table S2 in Multimedia Appendix 1). In short, key suggestions include creating the option to add personal tips more accessible, improving the app’s visual appeal and layout, simplifying questionnaires and activity choices, and adding new features like a planning tool, crisis plan, stress-relief methods, and a read-aloud function.

#### SAFE App

Together with clinicians, potential alterations were explored. Clinicians found the SAFE app user-friendly and appreciated its calming design, clear language, and wide range of methods to support individuals who self-harm and their relatives. Suggestions to improve the SAFE app included improving navigation, refining language (eg, using person-first terms), and adding disclaimers to emphasize that the app is not a replacement for professional help (Table S1 in Multimedia Appendix 2). In addition, based on the interviews and tests, we explored and identified a set of recommendations, which could improve the SAFE app for use in the Netherlands (Table S2 in Multimedia Appendix 2). In short, clinicians suggested adding culturally relevant distraction methods, replacing Danish videos with Dutch ones, and improving navigation and content organization. They also requested more actionable information for relatives, especially in crisis situations, and favored embedding content within the app instead of using external links.

### Step 4: Prototype

The prototype phase transforms ideas into tangible models to be tested and iterated upon. This phase is vital, as it enables us to explore and refine potential solutions through direct user interaction in interviews, workshops, or focus groups. Building prototypes allows one to quickly identify what works and what does not, gathering insights on how users engage with the solution and where improvements can be made. Key activities in this phase include creating solution prototypes that can be tested and refined through several iterations. Low-fidelity prototypes are quick, simple, low-cost representations, such as sketches or paper mock-ups. High-fidelity prototypes are more detailed and interactive, resembling the final product.

#### Low-Fidelity Prototypes and App Translation

##### SAM App

We conducted an A/B test, an experiment to test multiple versions of a solution, with 2 versions of text excerpts of translated app content, and finally, we performed a usability test of a prototype. To do so, we used an interview guide, including questions such as “Do you experience that individuals with autism have challenges with stress in their everyday life?,” “How do they deal with these challenges of stress now?,” and “Which tools do they have access to?” for part 1. This interview lasted approximately 90 minutes and was organized at the premises of the participants in order to encourage participation. The group interview was moderated by 2 project members (SLN and CKC), who shared the responsibilities of note-taking and moderating the dialogue. During part 2 of the group interview, the clinicians were presented with excerpts of draft content on paper in an A/B test to determine the tone and style of the language used. To do so, we had prepared a low-fidelity, paper-based prototype with 2 versions of the same content from the SAM app written in informal and formal language to test with the clinicians. We had translated parts of the app’s content in 2 versions to use during the interviews. One version was professionally translated, staying true to the original Dutch wording. The other version was translated by the project members by using both ChatGPT (Open AI) and a dictionary and in-house communication expertise and resulted in more informal wording. The formal version was thus a direct translation from Dutch, whereas the more informal version was altered in terms of writing style and readability, making the sentences shorter and using more layperson’s terms. All clinicians were handed a copy and got 5 minutes to review it and add their feedback. Afterward, we interviewed them in the group to elaborate on their feedback.

##### SAFE App

Once the need for a solution, such as SAFE was confirmed, the app was cloned to create a Dutch version (SAFE NL) to be used during the interviews and usability tests. The translation process was an iterative process, conducted in parallel with the remote focus groups via videoconferencing. The first translation from Danish to Dutch was conducted using a paid DeepL Translate (DeepL). After this initial translation, a member of the SUPER project team (JS) reviewed it for errors. Initially, only some parts of the app content meant for mental health practitioners were translated, in particular the section providing a definition of self-harm and the section on advice for clinicians, in order to get first feedback on the quality of the translation and to be able to adjust the translation plan if needed. In the second session, we presented the clinicians with a low-fidelity prototype. This document with the translated sections of textual app content allowed us to gather feedback on the translation quality on the one hand and on the usefulness of the app features on the other hand. The third session focused on explaining the artificial intelligence–driven translation process (using DeepL) to create a Dutch version of the SAFE app, followed by a review of the content using a high-fidelity prototype set up in Figma [33] to ensure cultural appropriateness and accuracy for use in the Netherlands. After having explored the prototype, clinicians were asked to provide both a positive point and a negative point about the app content, to provide feedback on the language used, and to say whether they would recommend the mobile app to clients, and they received the opportunity to request additional content.

### Content Adaptation

#### SAM App

Based on the feedback and input from the clinicians, the content of the SAM app, that is,translations and proposed activities was altered before actions with patients took place (Table S3 in Multimedia Appendix 1). In short, clinicians prefer shorter, more concrete, and informal language and appreciate the app’s scientific foundation. They suggested improvements, such as more detailed stress-reducing activities, shortening the questionnaire, and integrating the app into clinical practice rather than using it as a standalone tool. The questionnaire was not altered, as we prioritized the use of a validated questionnaire. We decided to use shorter sentences and more layperson’s terms and elaborate on activities and suggestions. After these alterations, the adapted content was transformed into a fully functioning prototype for testing with the intended users (patients).

#### SAFE App

Considering the translation process for the SAFE app was challenging, content changes were mainly focused on the language used and not the app content.

### Step 5: Test

The test phase focuses on evaluating and refining ideas or prototypes by seeking direct feedback from users through observing their interactions and gathering insights to identify strengths and areas for improvement. Testing ensures that the solution aligns with users’ needs and enhances its impact and usability. Key activities include testing the prototypes through several iterations and applying different methods. Early iterations with A/B tests or low-fidelity prototypes can provide valuable insights into the preferences of the intended users. An A/B test is used to compare multiple versions of a solution against each other to determine which one performs better. Later iterations can focus more on high-fidelity prototypes using usability testing and gathering feedback through interviews, surveys, and observations, evaluating how users interact with the solution. Usability testing can involve different methods, for example, user interviews, observations, and think-aloud tests, where users complete specific tasks while sharing their thoughts. This can help to understand how users engage with prototypes. Think-aloud tests encourage users to verbalize their thoughts as they navigate the prototype, which can reveal hidden challenges or unmet needs. By documenting findings systematically, testing becomes a valuable tool for validating assumptions and ensuring the solution is relevant and meets the intended user’s needs.

#### High-Fidelity Prototypes

##### SAM App

We used high-fidelity prototypes setup in Figma [33] on a laptop for a usability test, allowing focused feedback from clinicians to refine the Danish version of the SAM app. The clinicians were informed that the high-fidelity prototype used the direct translations from Dutch and involved a basic prototype of the app with limited functionality, meaning that not all functions were clickable. They were asked to explore the prototype for 2 minutes on their own before being tasked to navigate to specific parts and functions of the prototype. This included finding a tip for handling stress, filling out the SAM stress questionnaire, and generally clicking through the different functions and content of the app. This was followed by multiple interview questions: “How did you experience using the app?”, “Is the questionnaire manageable?”, “Is the purpose clear?”, “How did you experience the language in the app?”, and finally, “Based on your personal experiences and the brief review of the app, do you think that people with autism can benefit from the app for monitoring and handling stress?” In addition to user tests with clinicians, we also conducted usability tests with individuals with autism to observe how users interact with the app. Six individuals with autism tested the translated and adapted app on an iPhone (Apple Inc) to identify translation errors and usability issues, assess user satisfaction, and understand the app’s relevance in everyday use. Participants were asked to complete specific tasks within the app while their actions, reactions, and feedback were noted down. Specifically, and similarly to the actions performed with the clinicians, participants were first asked to explore the app and then asked whether the app’s purpose was clear and whether the app’s content and use made sense to them. Second, they were instructed to fill in the SAM app’s stress questionnaire and consequently asked, “Do the questions and answer options make sense to you?” and “How are the formulations?” Finally, they were tasked to navigate to specific parts and functions of the prototype and perform some exercises. These included finding a tip for handling stress, adding a personal tip to the app, and adding a note to the app’s diary function. This was again followed by multiple interview questions: “How did you experience using the app?” and “How did you experience the language in the app?,” and finally, “Based on your personal experiences and the brief review of the app, do you think that people with autism can benefit from the app for monitoring and handling stress?” The lessons learned from these interviews and usability tests are listed in Table S4 in Multimedia Appendix 1. In short, individuals with autism generally appreciated the app’s intuitive design and clear and concise language and found the stress questionnaire and predefined tips useful, although some found the questionnaire overwhelming and had difficulty locating specific features.

##### SAFE App

After having conducted 3 focus group sessions with clinicians, the fourth and final session provided the clinicians with a final status update on the translation process. Later, they received a final questionnaire to provide feedback on the app’s usability and specific features, for example, the type of exercises and the way exercises were presented. During this session, we also prepared the invitations and the testing schedule for relatives and clients to participate in testing the app together with the clinicians, as they were (rightfully so) very protective of their clients. Considering that the clinicians were not willing to provide direct access to their clients for user involvement, it was agreed that instructions were provided to the clinicians and that they subsequently conducted the usability tests with their clients. An overview of the lessons learned about the SAFE app from these activities with the clinicians is found in Table S1 in Multimedia Appendix 2. In addition, as a result of the lack of direct client access, we were only able to collect feedback from 1 individual with lived experience and 2 relatives through a web-based questionnaire. This questionnaire included open-ended questions, such as “What is your first impression of the SAFE app?” and “What can be improved about the SAFE app?” and close-ended questions, such as “What do you think about the various exercises in the app?” with answer options: “It is nice that there is a variety,” “I don’t need exercises,” and “I am missing exercises in the app.” Feedback from the clients and their relatives was thus mainly delivered by the clinicians (Table S3 in Multimedia Appendix 2). We were, however, reassured by the clinicians involved that they had effectively conducted the usability tests with their clients and their relatives but simply had failed to properly collect the associated data. Although we aimed for more direct involvement of these intended users (and advise to strive for this), our own pilot unfortunately lacks the required time and resources to do so.

## Discussion

### Overview

As design thinking is more a mindset than a framework, its application in practice is never exactly the same. Although both project partners (GGz Centraal and the Centre for Digital Psychiatry) relied on design thinking for the transnational adaptation of existing mobile apps, the performed steps and activities differed due to app-related variation, differences between target groups, and differences in background of the project partners. With this tutorial, we therefore aimed to illustrate how design thinking steps could be adopted in 2 real-life cases and demonstrate that even in countries with a quite similar culture (Denmark and The Netherlands), there can be a lot of variation in client preferences that might influence user engagement as well as the eventual uptake of digital mental health interventions.

### Common Themes in Transnational Mobile App Adaptation

Although both presented cases are very different, for example, mental health apps with a different goal (stress management vs providing advice and guidance in the context of self-harm), intended for different populations (people with autism vs people who self-harm and their next of kin and professionals), with a different technological system (translated version within the same app, SAM vs translated copy or the original app), there seem to be some transnationally relevant recurring themes and commonalities that might prove to be inherent to transnational scaling of mobile mental health apps. First, in both cases, the translation process proved to be time-consuming due to frequent alterations based on feedback from different stakeholders. In neither example case was it possible to create a direct translation (in 1 iteration) that used the correct terminology and tone of voice, according to the different stakeholders. Second, as mental health care systems and methodologies often vary across nations or sometimes even regions, mental health care professionals in both example cases requested changes to the app’s mental health content to fit their mental health customs and system better. For example, Danish mental health care professionals advised a shorter SAM questionnaire, more in line with their methods, while the Dutch professionals suggested other distraction methods and examples, more in line with their customs.

Finally, although both apps have initially been cocreated with various stakeholders and have been assessed in multiple studies, usability assessments always seem to reveal certain other preferences concerning the looks and feel of an app.

### Key considerations

When adapting a digital mental health solution for implementation and use in another country, there are several critical factors, aside from mere translation, to keep in mind to ensure that the solution is culturally relevant, accessible, and effective. Textbox 1 shows 9 considerations and recommendations when relying on design thinking for your adaptation, testing, and upscaling to a new market based on the implemented design thinking processes described in both examples and the lessons we learned. The first 2 recommendations are prerequisites for starting a transnational digital mental health initiative, namely ensuring sustainable financing and regulatory compliance; the following 7 are derived from our own experiences during this design thinking process. In short, we advise conducting thorough market research and involving local partners to assess (beforehand) whether upscaling to this new region is relevant and viable. From a practical perspective, the translation process should not be underestimated due to both cultural preferences and potential technical difficulties. In addition, design thinking should be considered as an iterative process, requiring flexibility and sufficient time from the designers. From an ethical perspective, it is important, as designers, to be open-minded, to communicate clearly about expectations toward the intended users, and to create a safe space for users to be involved in the design process.

Textbox 1. **Key considerations for using design thinking for international adaptation of digital mental health solutions.**

**Ensure sustainable financing**

It is crucial to have sustainable funding and a solid business plan from the beginning of the design thinking process. Uptake of even the best-designed product may fail without proper financial backing, as no resources will be available for maintenance, further development, or long-term sustainability.

**Ensure regulatory compliance**

Familiarize yourself with the local rules and legislation concerning digital mental health interventions in the target country to have a realistic view of not only the feasibility of your endeavor but also potential additional costs. Depending on your digital mental health solution, different laws will apply, for example, the European General Data Protection Regulation (GDPR), Medical Device Regulation (MDR), or, in some cases, the EU AI Act.

**Know your market**

Start by gaining an understanding of the target country’s mental health landscape, cultural norms, values, attitudes, and readiness toward digital mental health solutions. Do so by conducting an online or paper literature search, but also by reaching out to local experts. Understand which solutions and services are already available, what is lacking, and how your solution can meet this need.

**Collaborate with local experts**

Collaborations with local mental health organizations, professionals, and community leaders are challenging to set up without a pre-existing network. Their insights can nevertheless help navigate nuances in language and culture, regulatory requirements, and accessibility challenges. Collaborating with a local entity can also facilitate access to intended users for recruitment in various design thinking activities, such as interviews or usability testing.

**Apply design thinking as an iterative process**

Design thinking for user participation is more a mindset than a set framework. The main idea is to continuously test, evaluate, and refine your solution, sometimes requiring going back and forth through the phases. This flexibility is essential to develop an engaging, user-centered solution.

**Be open-minded and consider cultural, socioeconomic, policy, and other differences**

Cultural differences and similarities can appear in various ways. Be mindful of mental health perceptions and stigma, and create inclusive and diverse content, for example, visuals. Collaborate with local experts to align your solution with the cultural context and sensitivities of the target context and involve intended users to ensure clarity and relatabillity.

**Do not underestimate the translation process**

Despite the availability of translation supported by artificial intelligence, translating and adapting the content to the local context can be time-consuming and costly. Allow sufficient time for this task, as this will be a process rather than a single step. Frequent alterations might be required as you get feedback from different stakeholders moving through the design thinking steps.

**Communicate clearly with the involved users and clarify bidirectional expectations**

Set clear expectations by detailing the nature, duration, and frequency of the design thinking activities and what the user’s role is. In your dialogue, clearly state the added value of their input and the boundaries of their decision-making power and influence on the final solution. If feasible, it is also recommended to show appreciation for their participation and engagement by offering small tokens of appreciation or rewards after their efforts.

**Prioritize safety and flexibility**

When working with vulnerable groups, be mindful of ethical guidelines and create a respectful and safe environment for user participation. Local experts can be consulted for advice on how to approach and communicate with certain intended users. In addition, be flexible and pay attention to how comfortable they are with certain activities or topics.

Finally, the resulting guidelines [34] can be found on our Interreg North Sea project website [20]. The document titled “Guidelines for user participation: adapting digital mental health across borders” provides a practice-oriented, step-by-step guide for involving users in the development and adaptation of digital mental health solutions based on the principles of design thinking. Whereas this tutorial provides a description of how the design thinking activities were performed in 2 real-life cases, the guidelines document provides a summary of the design thinking steps, concrete tools, checklists, and methods to foster meaningful, respectful, and effective user participation.

### Limitations

Although design thinking is not a set methodology, this tutorial based on the design thinking framework does present a few limitations. First, the different design thinking steps have been performed with rather small samples. Although perspectives of even the smallest samples are relevant when involving intended users and other stakeholders in the digital mental health development and adaptation, larger, more varied groups might have provided more or other insights or clearer priorities for local app adaptation, for example, concerning different age groups or severity of conditions. In addition, in the adaptation of the Danish SAFE app to the Dutch context, we were not able to directly involve the intended users, namely people who self-harm (individuals with lived experience). Moreover, the design thinking process in both cases might have been influenced by selection bias due to samples that were not representative of an entire group. Second, despite the fact that this tutorial is meant for guiding the transnational adaptation of all types of digital mental health solutions, both presented example adaptations have been performed with mobile mental health apps. Other technology-specific issues not covered by this tutorial might occur when adapting other technologies, such as web-based platforms, virtual reality, or text messaging. Third, after transnational adaptation, the mobile apps were not assessed anew on effectiveness or efficacy in the new local context. Although no aspects of the apps’ working mechanisms were altered, it might be the case that through changes in wording, providing different exercises, or changing content, the app might not be as effective in the new population. We, therefore, advise reassessing effectiveness during the adaptation process if timing and budgeting allow.

### Conclusion

Digital mental health solutions have great potential to enhance mental health care. Involving users in the design, adaptation, and implementation process has been put forward as a potential solution; however, instructions and examples on how to do so are limited. This paper describes the steps of design thinking and how these design thinking steps can be undertaken by researchers, practitioners, or developers in the context of digital mental health, illustrated with 2 examples executed by the SUPER consortium in the Netherlands and in Denmark. The learnings from these 2 pilots are provided in the form of key considerations and highlight the issues that were experienced by the authors during both design thinking processes and strive to guide practitioners, developers, and researchers toward developing and internationally adapting better digital mental health tools in the future. This tutorial provides developers, academics, and clinical workers aiming to transnationally scale their digital mental health solution with guidance and concrete examples of why and how design thinking can be used to do so. Future research should focus on projects involving larger, more varied stakeholder groups to inform digital mental health app adaptation. In addition, both clinical practice and the research field would benefit from examples and evidence on transnational adaptation of digital mental health solutions to more varied local contexts beyond the Western population, for example, exploring Asian, African, or Middle Eastern populations.

## Supplementary material

10.2196/77048 Multimedia Appendix 1 Results and lessons learned concerning the adaptation of the Dutch Stress Autism Mate app for use in Denmark.

10.2196/77048 Multimedia Appendix 2 Results and lessons learned concerning the adaptation of the Danish SAFE app for use in The Netherlands.

## Abbreviations

SAM: Stress Autism Mate
SUPER: Successful User Participation Examples and Recommendations
