# Supplementary material for: A Design Thinking Approach for Transnational Adaptation of 2 Mobile Mental Health Apps: Tutorial for Researchers and Practitioners
Source: J Med Internet Res. 2025 Sep 17;27:e77048. doi: 10.2196/77048 (PMC12443347; doi:10.2196/77048)
Supplement: Multimedia Appendix 1 [file jmir-v27-e77048-s001.docx]

**Multimedia Appendix 1: Results and lessons learned concerning the adaptation of the Dutch Stress Autism Mate app for use in Denmark**

**Table S1.** Overview of the goals, needs, challenges and dreams of a fictional Danish individual with autism (persona), in relation to the Stress Autism Mate app, to help designers empathize with their target users.

| **Goals** | **Challenges** |
| --- | --- |
| 1. To reduce stress and handle stress better 2. To not be inhibited by stress 3. To have a better quality of life (to do what makes me happy) | 1. To maintain focus on the use of the app 2. To be able to use the app without help 3. To understand the unstated |
| **Needs** | **Dreams** |
| 1. Specific instructions and concrete examples (clear structure) 2. Short and clear formulations 3. Goal-oriented content, no unnecessary content. | 1. To be part of society on an equal basis as everyone else 2. To have a good quality of life (with their diagnosis) |

**Table S2**. Overview of the recommendations for alterations to the Dutch Stress Autism Mate app for use in Denmark based on the interviews and usability tests with both individuals with autism and clinicians.

| **Recommendation** | **Explanation** |
| --- | --- |
| Make the feature of adding own tips more easy to find | - Several participants struggled with adding their personal tip, because they could not find it in the menu or the settings. Based on observations during the usability test, there were two possible solutions. 1. Since the tips page was the first place many participants searched, this feature can be highlighted there. 2. Since several participants did not notice the information button on the top of the screen that states how to add a personal tip, the button can be changed to a more noticeable one. |
| Make the app lay-out more attractive by adding more colours and optimizing lay-out | - Several participants indicated that the SAM app was dull in its colour scheme, requesting more colours or contrast both in the app and for the app icon. Specifically, it was requested to highlight the bottom menu and clearly indicate the current page with a different colour. Furthermore, some participants indicated that the header, text and top buttons do not stand out, resulting in them going unnoticed by some participants. Accordingly, it was suggested to highlight headers and top buttons with a different contrast colour. - The use of colour was requested to clearly illustrate when they had chosen more options than allowed, both in the daily questionnaire and in the tip preferences, in order to not have to revisit their chosen options (instead of an error message). - A participant suggested optimizing the lay-out of the diary function so the calendar was hidden unless actively opened, in order to create more room for note taking. |
| Provide fewer answer and activity options in the questionnaire | - Both clinicians and individuals with autism suggested to make the survey more manageable by using a three-point instead of a five-point scale, since they were used to the shorter format. Additionally, some individuals with autism found the number of activity options overwhelming and suggested either fewer options or to have them organized in categories. |
| Potential new features | - A planning tool (e.g., to-do-list) to create a personalized plan to manage their stressor. For example, one participant also requested a crisis emergency plan to manage stressful situations, including who to call and what to do. - Stress relieving methods. Several participants expressed the need for a single app with everything they need to manage their stress. - A read-out-loud feature, to make the app more inclusive (e.g., for dyslexic users). |

**Table S3.** Overview of the lessons learned about the Stress Autism Mate app from interviews and usability tests with clinicians.

| **Topic** | **Summary of participant feedback** |
| --- | --- |
| Language | Preference for more concrete and shorter formulations of the text in the app as well as a more informal tone. |
| Evidence-base | Appreciation of the scientific, evidence-base for the app content, stressing as a good selling point for their clients. |
| Activities | Request for more specific and elaborately explained activities and tips to handle stress, eg, ‘find your shoes and put them on to go for a walk’ instead of ‘go for a walk’. |
| Questionnaire | Worries about the length of the in-app stress questionnaire as well as the number of answer options, seen as they use fewer in their practice. |
| Implementation | Suggestion to implement the application together with clinicians rather than as a stand-alone mobile application. |

**Table S4**. Overview of the lessons learned about the Stress Autism Mate app from interviews and usability tests with individuals with autism.

| **Topic** | **Summary of participant feedback** |
| --- | --- |
| Need for a stress tool | All participants had experienced significant challenges with stress throughout their lives and had developed tools and methods for managing their stress levels. They, however, believed that they could benefit from a tool such as SAM. |
| App design | Participants valued the SAM app’s user friendly and simple design. The structure feels intuitive. |
| Language | Throughout the user tests, everyone was very positive about the language, in particular appreciating to have short and precise sentences. Additionally, we addressed a potential challenge from the persona of understanding the unstated by concretising parts of the text. Throughout the test, some errors and mistakes related to the content and functionality have been continuously corrected as they have been found. Therefore, errors identified in a prior test may have been corrected before the next test. |
| Features: tips | Useful to have an overview of all potential tips and the option to choose your own tip from a predefined list. Available tips were sufficiently relevant. It was, however, challenging to add an individual tip as they could not find this option easily, though it was positively received that this option was available. |
| Features: questionnaire | The questionnaire was well received, its purpose was clear to the participants and could be completed without severe issues. However, some participants found the questionnaire overwhelming due to the many answer options.  Most participants also appreciated the stress level visualization (a color-coded indicator which they recognized from other contexts). |
