# Supplementary material for: A Design Thinking Approach for Transnational Adaptation of 2 Mobile Mental Health Apps: Tutorial for Researchers and Practitioners
Source: J Med Internet Res. 2025 Sep 17;27:e77048. doi: 10.2196/77048 (PMC12443347; doi:10.2196/77048)
Supplement: Multimedia Appendix 2 [file jmir-v27-e77048-s002.docx]

**Multimedia Appendix 2: Results and lessons learned concerning the adaptation of the Danish SAFE app for use in The Netherlands**

**Table S1. Overview of the** lessons learned about the SAFE app from interviews and usability tests with clinicians.

| **Topic** | **Summary of participant feedback** |
| --- | --- |
| Need | - Clinicians have individual preferences for tools and methods to provide clients information and guidance on self-harm, for example, a self-created list of 33 alternative actions to self-harm, mobile applications such as *Calm Harm* (<https://calmharm.stem4.org.uk/>), *Crisis Buddy* (<https://crisis-buddy.bd.aptoide.com/app>), and *DistrACT* (<https://www.expertselfcare.com/distract/>). - They believed that the added value of the SAFE app lies in its potential to empower users by giving them a sense of control, supported by its self-directed features and comprehensive methods for distraction and providing trustworthy information.  Moreover, the availability and intended use of the SAFE app for relatives and friends was also a solution to a clear to provide relatives with evidence-based information. |
| App design | - All participants liked the app's user-friendly and simple design. They also liked the use of colour, as it was experienced as calming and relaxing, and found the images of the different sections visually appealing. Several pointed out that the app's structure seemed intuitive, and it was not difficult to use. |
| Features | - They appreciated the audio fragments, as well as the ease of scrolling through the different sections. They prefer a simpler way to navigate back to the homepage. |
| Language and terminology | - In general, clinicians found the language used in the app clear and easy to understand. They requested to change the term for one of the target groups from ‘self-harmers’ to ‘people who self-harm’ throughout the app. In addition, the entry point (button) for people who self-harm had to be changed from ‘self-harmer’ to ‘for myself’. |
| App content | - All clinicians expressed satisfaction with the variety of methods provided, appreciating the range of tools they could use and experiment with when working with clients experiencing the urge to self-harm. They also noted that both clients and their relatives found the extensive list of methods valuable. The list included exercises, explanatory videos, and links to apps, as well as games or puzzles to engage with. - The informative articles were generally well received, and most clinicians understood their purpose. However, they were critical of the content, with some noting significant overlap across the different articles and would have preferred a bit more variety. |
| Disclaimer or warning | - Clinicians suggest the addition of a message advising to seek help from a professional (in the Read-section) when someone is showing self-harming behaviours, and that the app itself is not sufficient as a stand-alone aid. |

**Table S2**. Overview of the recommendations for alterations to the Danish SAFE app for use in The Netherlands.

| **Recommendations** | **Explanation** |
| --- | --- |
| **Additional methods** | - Clinicians suggested additional distraction activities and methods that are more common in the Netherlands, eg, holding an ice cube, feeling a rubber band around the wrist. |
| **Videos better suited to Dutch context** | - Since the Danish SAFE app includes Danish videos, subtitles were added for the pilot as a means to adapt the videos to the Dutch context. However, creating entirely new Dutch videos was considered a better solution and would have allowed them to be more relatable. |
| Categorize the informative articles | - Grouping the articles into different topics such as ‘coping strategies’, ‘self-harm’, ‘mental health resources’ would improve navigation and make it easier for users to find relevant information. |
| Optimize navigation to homepage | - A simpler way to navigate back to the homepage would improve the overall user experience by making it quicker and easier for users to return to the main menu without unnecessary steps. This is especially important in high-stress situations, where users may need to quickly access key sections of the app. Streamlining this navigation ensures a smoother, more efficient experience, reducing frustration and helping users stay focused on the support they need. |
| Additional information aimed at the section for relatives, specifically dedicated to providing help in a crisis situation | - Actionable steps for a crisis situation: Relatives indicated that they did not always manage to find the information they wanted, namely specific, actionable steps for what to do in a crisis situation. - How to seek help and support for oneself in a non-crisis situation. - Intended users, both relatives and those with lived experience need to be consulted anew in order to provide more actionable information. |
|  |  |
| Embed information and exercises in the app, instead of providing links leading away from the application | - Integration of in-app exercises, instead of linking to exercises outside of the app, will keep users more engaged. This also enhances privacy and security, as all the support resources are contained within a single, trusted environment. |

**Table S3**. Overview of the lessons learned about the SAFE app from interviews and usability tests with one individual who self-harms and two relatives of people who self-harm.

| **Topic** | **Summary** |
| --- | --- |
| App design: lay-out | - Users prefer a more clear distinction between the user profiles, eg, the colors of the buttons to choose your entry way (for myself vs for myself, for relatives and for clinicians). - Users preferred a more consistent and repetitive lay-out across the platform, eg, instead of a different lay-out and look on all of the ‘Try’-pages. |
| Language | - Users indicated that wordings and language is tailored to the target population, eg, different for youngsters vs adults. |
